# Rapid Identification of Phospholipase A_2_ Transcripts from Snake Venoms

**DOI:** 10.3390/toxins11020069

**Published:** 2019-01-25

**Authors:** Ying Jia, Pablo Olvera, Frida Rangel, Bianca Mendez, Samir Reddy

**Affiliations:** 1 Biology Department, The University of Texas Rio Grande Valley, Brownsville, TX 78520, USA; pablo.olvera01@utrgv.edu; 2 Mathematics and Science Academy, The University of Texas Rio Grande Valley, Brownsville, TX 78520, USA; frida.rangel01@utrgv.edu (F.R.); bianca.mendez01@utrgv.edu (B.M.); samir.reddy19@gmail.com (S.R.)

**Keywords:** snake venom, phospholipase A_2_ transcript, Bayesian inference

## Abstract

Phospholipase A_2_ (PLA_2_) is a major component in snake venoms and it is found in many different isoforms. To identify transcripts encoding different PLA_2_ isoforms, we developed a simple, rapid procedure. Total RNA was extracted from the venoms of three cottonmouth snakes and two diamondback rattlesnakes, and further reverse-transcribed into complementary DNA (cDNA). Using one pair of cottonmouth PLA_2_-specific primers and Reverse Transcription Polymerase Chain Reaction (RT-PCR) technique, we identified 27 unique full-length PLA_2_ transcripts, including nine sequences identical to the previously documented ones in the database and one novel GIII-like PLA_2_. Two common transcripts respectively encoding Asp49 and Lys49 PLA_2_ isoforms were identified in all three cottonmouth venoms that contain more PLA_2_ transcripts than diamondback rattlesnake venoms. The placement of cloned PLA_2_ transcripts in snake venom PLA_2_s was further discussed by phylogenetic analysis. The procedure developed in this study paves the way for accelerated acquisition of transcriptome data on any other venom toxin families. The results obtained are crucial for insight into the structure and function of PLA_2_ isoforms for scientific and potential therapeutic purposes.

## 1. Introduction

Phospholipases (PLs) are ubiquitous enzymes present in all organisms and organs, and their primary function is to hydrolyze phospholipids. Depending on which ester linkage of phospholipid is hydrolyzed, PLs are classified into PLA_1_, PLA_2_, PLB, PLC and PLD [1]. Among these PLs, PLA_2_s constitute a superfamily. Based on the primary structures and the organs to be expressed, PLA_2_s are further divided into 16 groups [2,3]. Snake venom PLs are a secretory PLA_2_ and belong to at least group IA and II: IA (Elapidae), II (Viperidae). Group II venom PLA_2_s are further divided into at least IIA (Asp49) and IIB (Lys49) types [4,5,6,7].

In addition to the primary function of hydrolysis of the 2-acyl bond of phospholipids releasing arachidonic acid and lysophospholipids, snake PLA_2_s display a wide variety of physiological activities such as neurotoxicity, myotoxicity, cardiotoxicity, platelet aggregation induction or inhibition, edema, hemolysis, anti-coagulation and hypotension [8,9]. The variety of different activities is due to the related plural PLA_2_ isoforms encoded by the multi-gene family generated by gene duplication events occurring over evolutionary time [10,11]. For example, *Protobothrops flavoviridis* (*Crotalinae*) group II PLA_2_ genes form a multi-gene family of 16–32 copies per haploid and are located at two loci on a microchromosome [12,13]. In addition, Shibata et al. [14] observed extensive duplication and accelerated evolution of venom genes such as PLA_2_ families.

Transcriptome analysis by means of the generation and random sequencing of venom gland complementary DNA (cDNA) libraries has proven to be an effective way of identifying novel transcripts of toxin proteins [15,16,17]. However, the major drawbacks of this approach include (1) the necessity of sacrificing snakes, (2) a complicated procedure (costly and time-consuming), and (3) sequencing too many redundant clones. Intriguingly, snake venoms contain only a few major protein families (PLA_2_, metalloproteinase, serine, C-type lectins, etc.) and plenty of gene information of these protein families exists in databases. Therefore, to identify unknown transcripts in specific protein families, it is necessary to develop a simple, rapid procedure. Additionally, a common characteristic of venom toxin genes is that the 5’- and 3’- end untranslated regions (UTRs) are more conserved than the protein coding region for a specific toxin family. Thus, the aim of this study was to utilize this characteristic and develop a fast, efficient and inexpensive approach for the rapid identification of full-length transcripts coding different PLA_2_ isoforms from crude venoms. The venom-based PLA_2_ transcripts obtained in this study can be used to explore the potential pharmacological activities derived from their counterpart venom proteins and offer important insight into the molecular evolution within the PLA_2_ multi-gene superfamily.

## 2. Results and Discussion

### 2.1. Cloning PLA_2_ cDNAs from Snake Venoms

**Figure 1:**
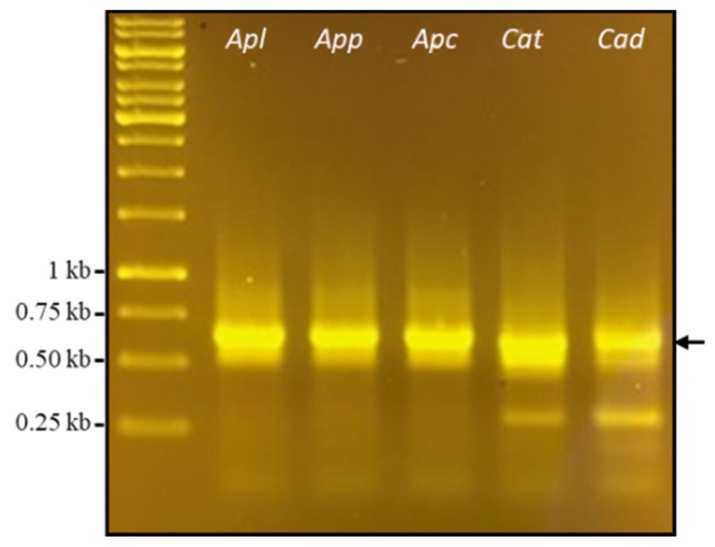
Reverse Transcription Polymerase Chain Reaction (RT-PCR) result. Snake venom phospholipase A_2_ (PLA_2_) complementary DNAs (cDNAs) were amplified from the reverse-transcribed cDNAs of each crude venom (*Agkistrodon piscivorus leucostoma*, *Apl*; *Agkistrodon piscivorus piscivorus*, *App*; *Agkistrodon piscivorus conanti*, *Apc*; *Crotalus atrox*, *Cat*; *Crotalus adamanteus*, *Cad*) using one pair of cottonmouth PLA_2_-specific primers. Arrow denotes the amplified PLA_2_ cDNAs.

**Figure 2:**
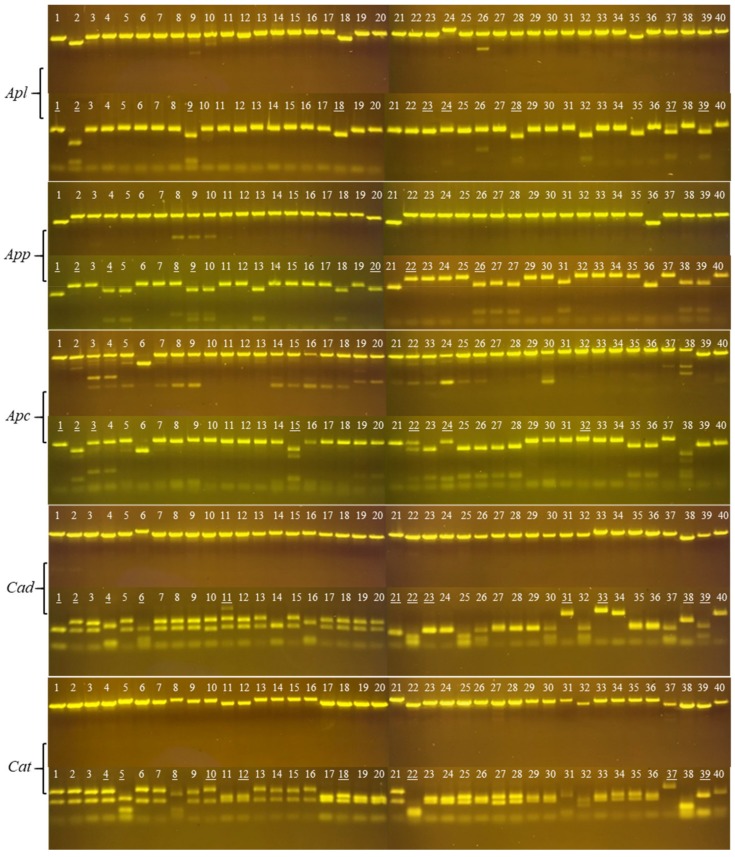
Selection of unique PLA_2_ transcripts. Polymerase Chain Reaction (PCR) amplicons (upper panels) were further digested by *Alu*I enzyme (lower panels). The clones with unique patterns (underlined) were subjected to sequencing.

To investigate PLA_2_ transcripts, we selected a related group of cottonmouth snakes (three subspecies of *Agkistrodon*), and a related species from a different genus (two rattlesnakes of *Crotalus*). These species were selected based on an abundance of PLA_2_ in their crude venoms, and the previous description of PLA_2_ transcriptome in venom glands and PLA_2_ toxins in crude venom [17,18,19]. PLA_2_ was chosen as the focus of this study because it constitutes a very large venom protein superfamily that exhibits various pharmacological activities. One of the distinctive characteristics of this venom gene superfamily is that the variation in the coding regions is higher than that in the non-coding regions for the same gene family [12,20,21,22]. Thus, we utilized this characteristic and transcript sequences we previously generated from cottonmouth snake glands to design two conserved primers located in 5’- and 3’- end UTRs of cottonmouth PLA_2_ transcripts. Figure 1 shows the Reverse Transcription Polymerase Chain Reaction (RT-PCR) result using cottonmouth PLA_2_-specific primers and template cDNAs reverse-transcribed from five venoms—*Agkistrodon piscivorus leucostoma* (*Apl*), *Agkistrodon piscivorus piscivorus* (*App*), *Agkistrodon piscivorus conanti* (*Apc)*, *Crotalus atrox* (*Cat*) and *Crotalus adamentus* (*Cad*). A strong band with the same size between 0.5 and 0.75 kb on five samples was successfully amplified after 35 cycles with a Polymerase Chain Reaction (PCR) annealing temperature of 55 °C. As we expected, cottonmouth PLA_2_-specific primers reacted strongly with the diamondback rattlesnake cDNAs (*Cat* and *Cad*), which further confirmed that a specific venom gene family in different species share conserved 5’- and 3’- end UTRs. After the gel purification of each of these bands, cDNAs were separately ligated into *Sma*I-digested pUC19 vectors by adding one unit of *Sma*I enzyme in a total ligation volume of 20 μL to prevent vector self-ligation, and we transformed each ligation into competent *Escherichia coli* cells by the heat shock method and obtained numerous white clones. We completed this process starting from crude venom to obtaining white colonies within 24 hours; using this procedure, cloning different toxin transcripts from crude venom is convenient, fast and economic. To our knowledge, since Chen et al. [23] cloned PLA_2_ cDNA from the venom of *Crotalus durissus collilineatus*, it has become a routine procedure to identify toxin transcripts from crude venom. Instead of a venom gland, using the crude venom (particularly the fresh venom) to obtain venom-derived mRNA possesses several advantages including (1) the same sample can be used for both transcriptomic and proteomic analyses, and (2) it avoids the necessity of sacrificing living snakes [24,25,26]. We randomly selected 80 white colonies for each ligation and screened for the expected molecular size (around 600 bp) of PLA_2_ full-length transcripts by PCR. Small molecules (less than 500 bp) were intentionally eliminated due to the possible 5’- or 3’- end fragments or immature transcripts. After eliminating short fragments, unamplified fragments and most of those with more than two bands, 40 clones with similar molecular weights for each venom (Figure 2, upper panel of each venom) were selected at the end for further restriction enzyme (*Alu*I) digestion (Figure 2, lower panel of each venom). Theoretically, enzyme *Alu*I with the recognition sequence AGCT will digest DNA frequently to produce an average size of 256 bp. From *Alu*I digestion patterns, it can be concluded that:

a) PLA_2_ transcript sequences are intraspecifically conserved;
b) None or one *Alu*I site is at the 5’- or 3’- end of cottonmouth PLA_2_ transcripts;
c) One *Alu*I site is near the middle of diamondback PLA_2_ transcripts.

**Figure 3:**
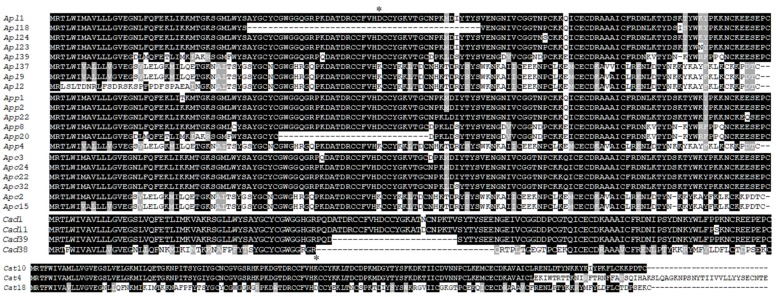
Amino acid sequence alignment of PLA_2_ isoforms. Multiple sequence alignment was carried out by CLUSTALW followed by BoxShade manual adjustments. * denotes the Asp49 (D49) or Lys49 (K49) type of PLA_2_.

Based on the molecular size and enzyme digestion patterns, a total of 45 clones with unique patterns (Figure 2, underlined clones) from all five snake venoms were selected for sequencing. All obtained cDNA sequences were annotated by a BLASTX search against the database (NCBI) to ensure that they were all full-length transcripts encoding PLA_2_. Each verified PLA_2_ cDNA sequence was further translated into a PLA_2_ amino acid sequence via the ExPASy translate tool (https://web.expasy.org/translate/). Based on the protein sequence, the missense mutations (at least one amino acid difference among PLA_2_ protein sequences) or frameshift mutations of PLA_2_ proteins were perceived as unique PLA_2_ isoforms in each snake venom. In the venom of *A. p. leucostoma* (*Apl*), nine clones (underlined clones) were selected for sequencing, resulting in eight unique clones (*Apl*1, *Apl*2, *Apl*9, *Apl*18, *Apl*23, *Apl*24, *Apl*37 and *Apl*39) (clones 9 and 28 were identical). In *A. p. piscivorus* venom (*App*), eight clones were selected for sequencing, resulting in six unique clones (clones 4, 9 and 26 were identical). In *A. p. conanti* venom (*Apc*), seven clones were sequenced, resulting in six unique clones (clones 1 and 22 were identical). Interestingly, *Apl*1, *App*2 and *Apc*24 encoding Asp49 PLA_2_ were identical, while *Apl*9, *App*4 and *Apc*15 encoding Lys49 PLA_2_ were identical (Figure 3). The deduced amino acid sequences of these two Asp49 and Lys49 PLA_2_s transcripts were the same as the amino acid sequences of PLA_2_ proteins (C0HKC3 and C0HKC2 in the database, respectively) purified from crude venoms [18]. These results further confirmed that cDNA sequences derived from snake gland transcripts can be used to identify unknown transcripts in crude venom for a wide diversity of the PLA_2_ protein family. Contrastingly, only seven unique PLA_2_ transcripts were identified from the diamondback rattlesnake venoms after sequencing 21 clones. From *C. adamanteus* venom (*Cad*), 12 clones were selected for sequencing, resulting in only four unique clones including two Asp49 full-lengths (*Cad*1 and *Cad*11) and two truncated ones (*Cad*38 and *Cad*39) (Figure 3, *Cad*); *Cad*1 and *Cad*23 were identical, while *Cad*2, *Cad*4, *Cad*6, *Cad*21, *Cad*22, *Cad*31 and *Cad*33 did not encode PLA_2_ proteins (they were non-coding regions). Nine clones were sequenced from *C. atrox* venom (*Cat*), resulting in only three unique clones including two Lys49 (*Cat*4 and *Cat*10) and one Asp49 (*Cat*18) (Figure 3, *Cat*), while *Cat*12 and *Cat*18 were identical, and *Cat*5, *Cat*8, *Cat*22, *Cat*37 and *Cat*39 were not PLA_2_ transcripts. Interestingly, *Cat*4 was a distinct PLA_2_ transcript similar to GIII PLA_2_ in length and to GIIB PLA_2_ in N-terminal sequence, but the sequence close to the C-terminus had a frameshift mutation leading to a long C-terminal tail (27 extra amino acids). The amino acid sequences of *Cad*1, *Cat*10 and *Cat*18 were identical with those of PLA_2_ with accession numbers (AUS82451, Q8UVZ7 and APD70896) in the database, respectively. Evidently, it is worth mentioning that (1) there are likely many more unique PLA_2_ transcripts in cottonmouth venoms than in diamondback rattlesnake venoms, and this result is comparable with the previous report [19], and (2) many other sequences including non-coding regions were incidentally amplified from diamondback venoms, implying that 5’- and 3’- end UTRs of diamondback venom PLA_2_ transcripts are slightly different from those of cottonmouth PLA_2_ transcripts.

### 2.2. Placement of Cloned PLA_2_ Transcripts in Snake Venom PLA_2_s

**Figure 4:**
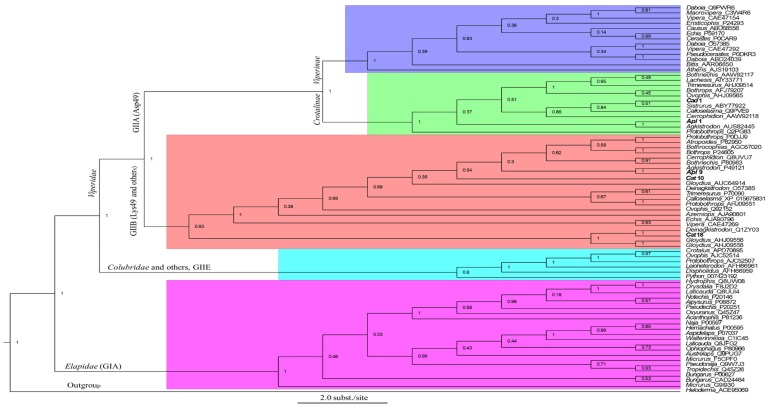
Phylogenetic analysis of snake venom PLA_2_s. The tree was constructed by the Bayesian inference (BI) method using Bayesian Evolutionary Analysis Sampling Trees, v1.8.4 (BEAST) based on aligned amino acid sequences. Most PLA_2_ amino acid sequences used were retrieved from GenBank and are represented by their genus name and accession numbers. Representative PLA_2_ isoforms obtained from this study are in bold. Numbers on branches are posterior probability values. PLA_2_ from a lizard (*Heloderma suspectum cinctum*, ACE95069) was used as a common outgroup.

Using the common amino acid sequence of Asp49 PLA_2_ transcript (*Apl*1 = *App*2 = *Apc*24) (Figure 3) present in all three cottonmouth venoms, we performed the protein BLASTX search against both non-redundant protein sequences (nr) and Swiss-Prot databases in NCBI on October 15, 2018, resulting in 761 snake venom PLA_2_ amino acid sequences from six families (Viperidae, Elapidae, Colubridae, Hydrophiidae, Lamprophiidae and Pythonidae), 47 genera and 167 species. Most PLA_2_ were Asp49 types, then Lys49 and others including Ser49, Asn49, Arg49, etc. The top four genera where most PLA_2_s were obtained, either in the form of PLA_2_ transcripts or native PLA_2_ proteins purified from crude venoms, are *Vipera* (140), *Trimeresurus* (75), *Crotalus* (71) and *Bothrops* (57). We individually performed an amino acid sequence alignment in each genus and selected one to three of the most conserved representative sequences (either Asp49 or both Asp49 and Lys49, depending the availability). Consequently, a total of 74 representative PLA_2_ amino acid sequences from 47 snake genera and one PLA_2_ (ACE 95069) from a venomous lizard, the Gila monster (*Heloderma suspectum cinctum*), as a common outgroup were used for the analysis of evolutionary relationships (Figure 4). Bayesian phylogenetic analysis of these PLA_2_s showed that different PLA_2_ types (e.g., GIA, GIIE, GIIB and GIIA) were clustered into the respective monophyletic group with strong confidence (posterior probability of 1.0), which supports the single early recruitment event for each PLA_2_ type before the separation of snake families. Specifically, all type IA PLA_2_s (GIA) were grouped into the Elapidae family, while type II PLA_2_s were clustered into the Viperidae family, which were further divided into GIIA (Asp49) and GIIB (Lys49 and others). Type IIA (Asp49) PLA_2_s were further distributed into paraphyletic subfamilies: Crotalinae and Viperinae. Meanwhile, all type IIE PLA_2_s from different families (such as Crotalidae, Viperidae, Lamprophiidae and Pythonidae) were grouped in the same clade with the strong support of statistical probability (>0.8). It was hypothesized that type IIA PLA_2_ genes may be evolutionarily derived from the IIE PLA_2_ gene, and then diversified into multiple gene types with several toxic activities such as myotoxic, hemolytic, edema-inducing and neurotoxic activities [27]. This prediction was further confirmed in this study, as all GII PLA_2_s were nested into group IIE PLA_2_s (Figure 4). As expected, Asp49 PLA_2_s (*Cad*1 and *Apl*1) obtained in this study from eastern diamondback rattlesnake (*C. adamanteus*) and cottonmouth venoms, respectively, were grouped into the *Crotalinae* superfamily; particularly the *Agkistrodon* PLA_2_s (*Apl*1) and AUS82445 (isolated from *Agkistrodon contortrix contortri*) were in the well-supported same group (1.0 posterior probability). Both Lys49 PLA_2_s (*Apl*9 and *Cat*10), cloned from western cottonmouth and western diamondback rattlesnake venoms, respectively, were grouped into GIIB PLA_2_ (1.0 posterior probability). However, the analysis here placed the basic Asp49 PLA_2_s (including *Cat*18 obtained in this study from *C. atrox*, Q1ZY03 from *Deinagkistrodon acutus* [28], AHJ09556 and AHJ09558 from *Gloydius* [29]) as a sister group to the strongly supported clade (posterior probability of 93%) of other basic GIIB PLA_2_ homologs. In comparison, the phylogenetic tree topology in this study is strikingly similar to those generated by Modahl et al. [26] and Shibata et al. [14], and the higher-level relationships of snake venom PLA_2_s across these trees are (GIA (GIIE (GIIA + GIIB))). However, relationships among the snake families of Elapidae, Viperidae and Colubridae differ somewhat between the tree here and those generated by Zheng et al. [30] and Pyron et al. [31], probably due to the use of different genes: Elapidae is sister to Viperidae and Colubridae in this study, whereas Elapidae is nested inside of Viperidae in the trees constructed by Zheng et al. [30] and Pyron et al. [31]. Overall, the phylogenetic tree generated on the basis of amino acid sequences of PLA_2_ clearly grouped snake families or subfamilies as distinct clades (Figure 4), therefore, PLA_2_s can be used as a venom toxin gene in inferring the evolutionary history of snake species.

Collectively, a total of 27 unique PLA_2_ transcripts were identified after sequencing 45 clones using a simple, rapid technique; these 27 transcripts included eight, six, six, four and three transcripts from the crude venoms of *A. p. leucostoma*, *A. p. piscivourus*, *A. p. conanti*, *C. adamanteus* and *C. atrox*, respectively. Transcripts encoding Asp49 and Lys49 PLA_2_ isoforms were identified from each venom except *C. adamanteus* venom, in which only Asp49 PLA_2_ was obtained. The placement of some of the obtained PLA_2_ transcripts in snake venom PLA_2_s was phylogenetically analyzed. Importantly, snake venom toxins are encoded by relatively few (approximately 5–10) multi-locus gene families, with each gene family possessing conserved 5’- and 3’- end UTRs. Therefore, the use of conserved primers to identify unknown full-length transcripts for each toxin gene superfamily can be employed to screen transcript sequences within each species for toxins of interest, and to examine novel mutations within a venom protein family. The established technique and results are readily accessible to many researchers and provide a basis for future studies identifying the unique full-length transcripts for any other toxin superfamilies in unexplored venoms.

## 3. Materials and Methods

### 3.1. Cloning PLA_2_ cDNAs from Crude Venoms

Five different venoms, extracted from three cottonmouth snakes (Agkistrodon piscivorus leucostoma, Agkistrodon piscivorus piscivorus and Agkistrodon piscivorus conanti) and two diamondback rattlesnakes (Crotalus atrox and Crotalus adamanteus) in July 2015 were purchased from Miami Serpentarium Lab (Punta Gorda, FL, USA). Ten milligrams of each lyophilized venom mixed with 1 mL of TRIzol Reagents (ThermoFisher Scientific, Waltham, MA, USA) was used to isolate total venom RNA. The isolated total RNA was further reverse-transcribed into complementary DNA (cDNA) using a Maxima First Strand cDNA Synthesis kit (ThermoFisher Scientific) according to the instruction manual. Reverse Transcription Polymerase Chain Reaction (RT-PCR) was performed using a pair of cottonmouth PLA_2_-specific primers (Forward: 5’-CCGGCTTCTCCTTCTGATCCTT-3’, Reverse: 5’-GAGTGCAAAGCTGGCACCTGT-3’) and Phusion High-Fidelity PCR Master Mix (ThermoFisher Scientific). PCR products were separated in 1% agarose gel and further purified using a GenJET PCR Purification Kit (ThermoFisher Scientific). SmaI enzyme-digested pUC19 plasmid DNA (BioLabs, Ipswich, MA, USA) was ligated with purified PCR product using T4 DNA ligase (ThermoFisher Scientific) at room temperature for 1 hour. The ligation was transformed into competent E. coli cells (DH5α) and then spread on LB (Luria–Bertani) agar plates containing ampicillin (75 μg/μL), X-Gal (20 mg/mL) and IPTG (100 mM). The plates were then incubated at 37 °C overnight. At least 60 white colonies were selected and incubated in each colony in 300 μL of LB broth containing ampicillin overnight at 37 °C. Overnight culture (10 μL) mixed with 40 μL TE buffer was boiled in hot water for 3 minutes, then centrifuged for 3 minutes at top speed. The supernatant (1 μL) was used as the DNA template for PCR using M13 universal primers located on pUC19 vector and Classic Hot Start Taq DNA Polymerase (Tonbo Biosciences, San Diego, CA, USA) in a total volume of 20 μL. The PCR product (10 μL) was used to examine the expected molecular size for PLA_2_ full-length cDNA (approximately 600 bp) on 1% agarose gel, and the other 10 μL PCR product was used to detect the different sequences for clones with the same molecular size by AluI (BioLabs) restriction enzyme digestion. Based on molecular size and enzyme digestion patterns, the unique PLA_2_ cDNAs were selected for Sanger sequencing at the University of Texas at Austin (Austin, TX, USA). The identity of transcripts was verified by comparison of the translated cDNA sequences with previously characterized PLA_2_s using a BLASTX search against both non-redundant protein sequences (nr) and UniProtKB/Swiss-Prot (Swiss-Prot) databases in the National Center for Biotechnology Information search database (NCBI). Signal sequences of each deduced PLA_2_ proteins were ascertained using the SignalP v4.1 server (http://www.cbs.dtu.dk/services/SignalP/). Amino acid sequence alignment was performed by CLUSTALW (http://www.genome.jp/tools/clustalw/), followed by BoxShade (http://www.ch.embnet.org/software/BOX_form.html) modification. All full-length sequences were submitted to NCBI GenBank (accession numbers MK393884-MK393901).

### 3.2. Phylogenetic Analysis of Snake Venom PLA_2_s

To determine the placement of cloned PLA_2_ transcripts in snake PLA_2_ proteins, all amino acid sequences of snake PLA_2_s were retrieved from both non-redundant protein sequences (nr) and UniProtKB/Swiss-Prot (Swiss-Prot) databases by blasting the deduced mature amino acid sequence of common Asp49 PLA_2_ cDNAs cloned from all cottonmouth snake venoms in this work, and by setting up an expected threshold of 10^−5^ and max target sequences of 1000, based on the general rule that sequences with an *E*-value less than 10^−5^ are homologs of a query sequence [32,33]. A phylogenetic tree of Bayesian inference (BI) was constructed using Bayesian Evolutionary Analysis Sampling Trees, v1.8.4 (BEAST) [34] through the following procedure. A strong multiple alignment of amino acid sequences with an average p-distance of 0.473 and a maximus pairwise p-distance of 0.68 [33] was acquired by performing the MUSCLE program on the MEGA7 package [35] followed by visual inspection for errors and saved as a Nexus format through Seaview v4 [36]. The Tracer (v1.7.1) program [37] was used to eliminate the early low-probability trees from the final consensus tree by setting up the burn-in value of 100,000. We produced maximum clade credibility (MCC) trees using TreeAnnotator [38]. A deduced amino acid sequence of PLA_2_ obtained from a lizard (*Heloderma suspectum cinctum*) (Account number: ACE95069) was used as a common outgroup because both the snakes and lizard were revealed to be members of a clade (Toxicofera) whose venom systems are homologous [39]. The resulting phylogenetic tree was visualized in FigTree v1.4.3 [40] and the rotation was manually adjusted.
